# The Effect of Positive and Negative Feedback on Risk-Taking across Different Contexts

**DOI:** 10.1371/journal.pone.0139010

**Published:** 2015-09-25

**Authors:** Annabel B. Losecaat Vermeer, Alan G. Sanfey

**Affiliations:** 1 Behavioural Science Institute, Radboud University Nijmegen, Nijmegen, The Netherlands; 2 Donders Institute for Brain, Cognition and Behaviour, Radboud University Nijmegen, Nijmegen, The Netherlands; University of Bologna, ITALY

## Abstract

Preferences for risky choices have often been shown to be unstable and context-dependent. Though people generally avoid gambles with mixed outcomes, a phenomenon often attributed to loss aversion, contextual factors can impact this dramatically. For example, people typically prefer risky options after a financial loss, while generally choosing safer options after a monetary gain. However, it is unclear what exactly contributes to these preference shifts as a function of prior outcomes, as these gain/loss outcomes are usually confounded with participant performance, and therefore it is unclear whether these effects are driven purely by the monetary gains or losses, or rather by success or failure at the actual task. Here, we experimentally separated the effects of monetary gains/losses from performance success/failure prior to a standard risky choice. Participants performed a task in which they experienced contextual effects: 1) monetary gain or loss based directly on performance, 2) monetary gain or loss that was randomly awarded and was, crucially, independent from performance, and 3) success or failure feedback based on performance, but without any monetary incentive. Immediately following these positive/negative contexts, participants were presented with a gain-loss gamble that they had to decide to either play or pass. We found that risk preferences for identical sets of gambles were biased by positive and negative contexts containing monetary gains and losses, but not by contexts containing performance feedback. This data suggests that the observed framing effects are driven by aversion for monetary losses and not simply by the positive or negative valence of the context, or by potential moods resulting from positive or negative contexts. These results highlight the specific context dependence of risk preferences.

## Introduction

We often make decisions that involve some level of uncertainty about what the final outcome will be, whether it be choosing which restaurant to pick for dinner, deciding to place a bet on who will win the World Cup, or thinking about whether to go on a blind date or not. In general, people dislike risky or uncertain outcomes [1, 2]. For example, when offered a choice between a certain €10, or the opportunity to play a gamble which pays either €20 or €0 with equal probability, the vast majority of people choose the certain €10 option. In fact, our preferences for risky choices have been well established as unstable and context-dependent. Whether or not we place a large bet to win the World Cup pool may depend on whether we have just won or lost money, even though the current bet is clearly independent of this previous outcome. Similar behavioural effects have been observed in many everyday scenarios, such as in trading [3], betting at racetracks [4], health decisions [5], as well as with clinical populations [6].

Research has shown that choices between a risky and a safe option made immediately after experiencing a financial loss typically lead to a higher preference for the riskier bets [2, 7, 8, 9]. For example, one experiment [8] showed that deciding to either play or pass on a gamble was influenced by the outcome of prior gambles. Participants decided to play the gamble more often following a loss on the previous gamble trial as compared to a win beforehand. In a previous study from our group, [10] we found individuals’ risk preference to be sensitive to a preceding monetary gain or loss. These gain and loss outcomes were based on correct or incorrect performance, respectively, on a time-estimation task. Importantly, these performance-based monetary gain/loss outcomes were unrelated and independent of subsequent gambles. Immediately following these gain/loss outcomes, individuals decided to either play or pass on a 50–50 mixed (gain-loss) gamble. We found that individuals chose the gamble more often after receiving the performance-based monetary loss than after receiving a performance-based monetary gain. In other words, outcomes independent of the decision at hand (e.g. recent gains or losses) can influence risk preferences in determining our choices, in systematic ways.

In the aforementioned studies, shifts in risk preferences following monetary gains and losses were also related to the outcome of players’ actions. In other words, outcomes were based on either the individuals’ decision to play a gamble previously [7, 8], or were based on the individuals’ actual performance on an unrelated task [10]. Hence, the observed behavioural effects on gambling behaviour following gain/loss outcomes could be related to either the monetary outcomes (gains and losses) or to the performance measures themselves (i.e. whether the task was successfully completed or not). In the current study we aimed to clarify whether gain/loss context effects are driven by monetary feedback or by performance-based feedback.

Pure monetary gain and loss outcomes have previously been shown to affect risk preferences. Susceptibility to pure monetary gains and losses have been most consistently observed for mixed gambles, that is, those offering a chance to both win and lose money, for example a 50/50 chance to either gain €50 or lose €50. People typically choose to avoid playing such gambles, even when the expected value is equal to or even higher than the option to not play the gamble. This behaviour has been explained by the concept of loss aversion in Prospect Theory (i.e. a relatively greater sensitivity to losses than to gains of equivalent value) [2, 11]. This also holds for choice outcomes that have been framed as either gains or losses of equal expected values. For example, given a choice between keeping €20 for certain from a €50 endowment, or the opportunity to play a gamble with a chance to keep the full €50 and a chance to get nothing (i.e. gain frame), people prefer the certain €20. Conversely, when offered a choice between accepting a certain loss of €30 from the €50, or a gamble with a chance of either losing the full €50 or of losing nothing (i.e. loss frame), people tend now to prefer the gamble (the so-called ‘framing effect’, [1]). Thus, pure monetary gains and losses affect risk preferences. However, whether pure monetary gains/losses independent of an active choice also affect risk preferences in a similar manner is as yet unclear.

Evaluation of received rewards and punishments typically informs us as to whether we should either continue or adapt our current behavioural strategy [12]). It is unknown however whether performance feedback *per se* influences subsequent risk in a similar direction as pure monetary feedback. There are some studies that have demonstrated preference shifts for financial risk following unrelated, non-financial, outcomes [13, 14]. For instance, incidental mood, induced via the viewing of valenced stimuli, has been shown to influence risk preferences for mixed gambles [13], as well as to enhance monetary framing effects [14]. Positively arousing stimuli (i.e. erotic pictures), unrelated to the gambling task itself, increased preference for a high-risk option, with importantly this effect not observed after viewing either neutral (i.e. household appliances) or negatively valenced pictures (i.e. snakes or spiders, [13]). Similar behavioural results were found for framing effects involving monetary gains or losses, with accentuated framing effects following positive film clips (i.e. inducing happiness) as compared to negative film clips (i.e. inducing sadness, [14]). These studies indicate that preference shifts for monetary risk are highly context-dependent and can be influenced by loss aversion, but also by mood effects. Performance-based feedback prior, but unrelated to, the risky choice might therefore also impact risk preferences in a similar vein to these unrelated, non-financial, outcomes.

Our current study directly compares performance and monetary contexts prior to, and independent of, identical mixed gambles. The aim of this study is to examine whether the context-dependent preference shifts that were observed for mixed gambles following performance-based monetary rewards and punishments [10] are purely driven by the receipt of monetary gains/losses, or if performance-based success/failure feedback, independent of monetary reward, can also be a factor in reframing the choice. This will provide a more thorough understanding of how risk preferences may be altered by different contexts, and specifically will yield a more comprehensive idea of the possible role of loss aversion across different contexts. To examine this question, participants were presented with identical sets of mixed gambles following different types of positive and negative feedback. We investigated three different types of contexts, all provided on a task unrelated to the gambles themselves: 1) monetary gain or loss directly based on participants’ performance as in our previous study [10], 2) a ‘pure’ monetary gain or loss that was randomly allocated, and crucially, was independent of any task-related performance, and 3) success or failure feedback based on the participants’ performance, but without any associated monetary gain or loss. Immediately following each of the aforementioned context types, participants decided to either play or pass on a mixed gamble which offered a monetary gain with a probability of 0.5, and a similarly-sized monetary loss with a probability of 0.5. If they chose to play, the outcome of the gamble would be then shown. If they decided to pass on the gamble, the next trial would immediately begin.

Based on prior work [10], we expected performance-based monetary gains/losses to influence risk preferences, that is, to lead to an increase in risk-taking following a monetary loss, and a decrease in risk-taking following a monetary gain. As predicted by loss aversion, we hypothesized that individuals would also increase risk-taking after receiving randomly allocated monetary losses versus similarly distributed gains. However, we expected that the behavioural effect observed for performance-based gain/loss outcomes might be stronger than after incidental monetary gain/loss outcomes. Monetary gains/losses that are earned (via successful performance) might enhance the sensitivity to losses (i.e. endowment effect, [15]) as compared to incidentally receiving monetary gains/losses. Furthermore, we explored whether performance feedback of success and failure influences risk preferences for monetary gambles. If performance feedback does not affect risk preferences, then it would clarify that the framing effects observed in the previous study are primarily driven by monetary gains/losses, and not, or at least less than, by other contexts, such as mood effects potentially induced by successful or unsuccessful performance [16].

## Materials and Methods

### Participants

A total of 75 students participated in the study. All gave written informed consent and either received research credits for participation or were financially compensated via a flat fee (10 Euro). They also had the opportunity to win a bonus based on their final experimental balance (up to a maximum amount of 10 Euro). Hence, participants’ total payment at the end of the experiment would range between €0 and €10 if they participated for credits, and between €10 and €20 if they participated for money. Exclusion criteria were self-reported regular drug use of marijuana, or use of psychotropic drugs. Three participants were excluded as they did not respond to a substantial number of trials (>20%). Data is therefore reported from 72 participants (Men = 21, *M* = 21.86 years, *SD* = 3.46). The study was performed in accordance with the Declaration of Helsinki and approved by the Ethics Committee Faculty of Social Sciences of the Radboud University (ECSW2015-1105-305). This approval covers behavioural testing that do not apply any invasive intervention (e.g. medication) and includes only healthy, legally competent adults (>18 years of age) as participants.

### Task design and procedure

We adapted a previous paradigm [10] in order to study risk-taking behaviour in the context of prior rewards and punishments of different types. The task contained three different experimental trial type conditions; monetary trials, performance trials, and combined performance/monetary trials. Each of these trials was designed to induce either a positive (reward) or negative (punishment) context.

In the monetary trials, either a positive (+ €1.20) or negative (- €1.20) monetary amount was randomly awarded on each trial. Each trial began with a pair of dice displayed on the screen. If the total sum of dots was between 7–12 participants would win €1.20, however, if the total sum of dots was 2–6 they would lose €1.20. The total sum of dots on the dice was randomly computed for each trial, to ensure approximately 50% wins and 50% losses.

In the performance trials, participants either successfully or unsuccessfully completed a simple time-estimation task in which participants received feedback based on their ability to estimate a temporal duration of a second. This was adapted from a previous paradigm [10]. Each trial began with a pair of red dice that changed colour to white. When the dice turned white, participants were then required to respond exactly one second later by pressing a key. If they responded within an allowable window then the trial was labelled a success, otherwise it was deemed a failure. The purpose of the time-estimation task was to induce either a positive or negative context, that is one based on performance and not monetary reward, to enable a test of whether subsequent shifts in risk behaviour are driven by task performance-related success or failure.

Finally, in the combined trials, participants received both performance feedback as well as monetary feedback. In this condition, we used the same paradigm that was for the performance condition, with the addition of monetary feedback. That is, if the time-estimation task was successfully performed participants additionally received a monetary reward (+€1.20) and if unsuccessful on the time-estimation task then participants took a monetary loss (-€1.20), identical to the original paradigm [10].

**Fig 1:**
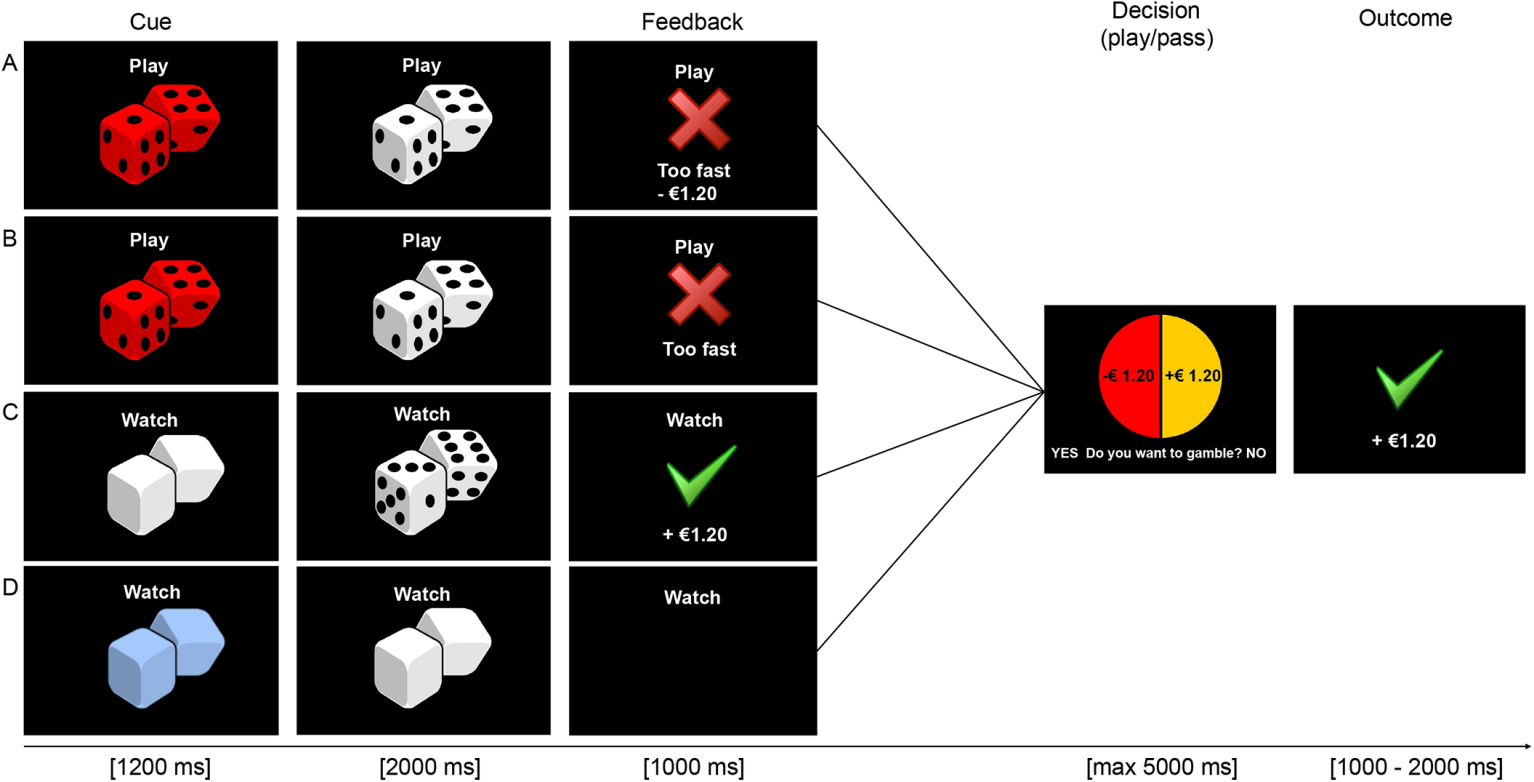
Task design. Each picture represents a screen in the experiment. A) The combined monetary and performance (MP) trial; Trial began with a time-estimation task, where participants were required to press a button exactly 1 s after the red dice changed in colour to white. Feedback on performance was given as “Correct” with a monetary gain of €1.20, and if incorrect as “Too fast” or “Too slow” with a loss of €1.20, B) The performance (P) trial; Trial structure was the same as A. However, only success and failure feedback on performance was given, as “Correct” and if incorrect as “Too fast” or “Too slow”, C) The monetary (M) trial; Trial began with a white cue that changed to a pair of dice. Participants only had to passively watch, as instructed on top of the screen. They were instructed that if the total sum of dots on the dice was higher than 6, participants gained €1.20, if the total sum of dots was 6 or less, the participant lost €1.20, D) Control trial; Trial began with a blue cue that changed in colour to white. Participants only had to passively watch, as instructed on top of the screen, no feedback or incentive was given. For all trial types, immediately following feedback or no feedback (i.e. D), participants had the opportunity to choose to play or pass on a mixed gamble with a 50/50 chance to either gain or lose money. If participants decided to gamble, the gamble was played and the outcome was presented. Average duration of a trial is 9–13 s.

In all the three trial type conditions described above, immediately following the positive and negative feedback contexts, participants were shown a mixed (50/50 chance, gain/loss) gamble (see Fig 1), which they could decide to either play or pass. If they decided to pass on the gamble the trial immediately ended. However, if they decided to play, the gamble was resolved and the corresponding win or loss amount was added to their total experimental balance. Participants’ running balance was not directly displayed during the experiment, but was only shown at the conclusion of the experiment. Participants had been informed that they would be paid this balance (if positive) as an experimental bonus.

The mixed gamble contained either a positive expected value (‘+ EV’), a negative expected value (‘- EV’), or a zero expected value (‘0 EV’), constructed by varying the gain or loss outcome from €1.00, €1.20, to €1.40 (see Table 1). We created these three different gamble types to assess whether participants were attending to, and sensitive of, the expected value of the gamble.

**Table 1:** Mixed gambles by expected value (EV) and EV type.

50|50 mixed gamble	Expected value (EV)	Gamble type
- €1.40 | + €1.20	-0.10	- EV
- €1.20 | + €1.00	-0.10	- EV
- €1.20 | + €1.20	0	0 EV
- €1.00 | + €1.20	0.10	+ EV
- €1.20 | + €1.40	0.10	+ EV

All gambles contained of a 50–50 probability to lose-win money

In addition to these experimental conditions, we included a control trial to enhance engagement in the task. Each control trial began with a pair of blue dice that changed colour to white. Participants were required to watch these cues and not respond, similar to the monetary condition. However, participants did not receive any feedback following these cues. After the presentation of the white cue, participants received a mixed gamble which they could decide to either play or pass, identical to the other three conditions as described above (Fig 1). Also, 12.5% of the total trials were “no-gamble” time-estimation trials. In these trials, participants were not presented with a gamble after receiving feedback on the time-estimation task. The no-gamble trials were indicated in advance by a specific visual cue (cubes instead of dice), and were employed to enhance engagement in the gamble trials (Fig 1). These trials were not included in the analysis, as they did not contain a gamble. Time–estimation performance on these trials did however affect the experimental balance, in the case of receiving a bonus.

### Procedure

Participants first performed two short practice sessions. In the first session participants practiced the time-estimation task. Here, participants were required to estimate a one-second time duration. After a cue on the screen changed in colour, they were instructed to wait exactly one second and then press a response button, with their precise response times recorded. We used the minimum and maximum of these recorded response times for each participant to determine an initial allowable response time-window for the performance-based trials in the experiment. The second practice session gave participants the opportunity to observe the gambling task and experience the different trial type conditions. After these practice sessions, the experiment lasted for approximately 30 min including a short break halfway through the task.

Before beginning the task, participants were instructed that their goal was to win as much money as possible, and that their final balance would be paid out as a bonus (with a maximum amount of €10). At the start of each trial, participants saw a visual cue with the instruction to either “Watch” or “Play”, to remind them whether they had to only watch the screen or perform a time-estimation task. When it said “Watch”, participants would see a cue change to either a different colour indicating a Control trial, or see a cue change to a pair of white dice, indicating a Money (M) trial. Depending on the total sum of dots on the dice in the M trial, participants won or lost money (see Fig 1). When the start of the trial said “Play” participants had to perform the time-estimation task, that is, depending on the specific cue participants would play a performance only trial (P) or a combined trial (MP), or a no-gamble trial (Fig 1). Responses on the time-estimation task were considered correct when they were within an allowable time-interval. For correct responses, participants received positive feedback either in the form of “correct” (performance trial) or in the form of “correct” including a gain of €1.20 (combined trial). When a response was not within this time-interval, participants either received negative feedback as “too fast” or “too slow” (performance trial), or received the same negative feedback with an additional loss of €1.20 (combined trial). The allowable response-interval of these trials was initially calculated based on their performance in the practice run and then covertly adjusted throughout the task as a function of the variance in response time of the participant in order to ensure an approximately equal number of positive and negative feedback on this task. If participants responded within the allowable response-interval, this interval was subsequently shortened by 50 ms; if they responded either too quickly or too slowly, the interval was then lengthened by 50 ms. Importantly, although the amount of positive and negative feedback was manipulated, the feedback was contingent upon participants’ performance. Thus, the time-interval was adjusted individually based on the participant’s actual response behaviour (see [17]).

Immediately following the feedback on both trial types, with the exception of the no-gamble trial, participants could choose to either play or pass on a mixed (50/50 chance, gain/loss) gamble. Playing the gamble led to two possible outcomes: 1) A win outcome which added either €1.00, €1.20 or €1.40 to their overall experimental balance, or 2) a loss outcome which subtracted either €1.00, €1.20 or €1.40 from this balance, depending on the type of gamble offered (see Table 1). Alternatively, the participant could decide to pass on the gamble, thereby keeping the earlier monetary gain or loss (i.e. +/- €1.20) in case of the M and MP trials. The gamble outcomes were independent from the performance on the time-estimation task and the outcome of the M trials. All gamble outcomes (both gains and losses) immediately updated the total running balance for each participant. This balance was not directly displayed to the subject during the experiment, but was only viewed at the conclusion of the task.

In total, participants played on average 140 trials (of which approximately 12.5% “no-gamble” trials). The design contained a nested structure. 75% of the trials were experimental gamble trials (*M* = 104, range = 91–115, with a mean number of trials per condition: M = 35, MP = 34, P = 35), that each contained approximately 50% positive feedback (*M* = 53 trials) and 50% negative feedback (*M* = 51 trials). Within each set of these positive and negative feedback, 1/3 of the trials had a negative EV gamble (*M* = 35), 1/3 a zero EV gamble (*M* = 34), and 1/3 a positive EV gamble (*M* = 35), all randomly presented. Furthermore, 12.5% of the total trials were “control” trials that did not contain feedback (*M* = 18), but contained the same distribution of the EV gambles. Note that the total number of trials were slightly unbalanced due to using a performance-based adjustment to determine an approximate equal number of positive and negative feedback outcomes during the task, that was still contingent upon the subjects’ real performance. Each trial in the game varied between 9–13 seconds. The task was presented in Presentation® software (Version 14, www.neurobs.com).

### Analysis

In order to assess the degree of risk-taking following positive and negative feedback contexts respectively, we assessed the number of gambles played/passed as a binomial dependent measure. We had three within-subject factors: ‘context type’ (three levels: Monetary (M), Performance (P), Monetary and Performance combined (MP)), ‘feedback context’ (two levels: Positive, Negative), and ‘gamble type’ (three levels: positive EV, zero EV, negative EV). The Control trials did not have positive or negative feedback and were not added to the main model. A generalized linear mixed effect model was performed, to avoid aggregation of the data, thereby maintaining intra-individual variance, and due to the data being slightly unbalanced in number of trials. For this, we used the *mixed* function of the package for Analysis of Factorial Experiments (afex, v.0.12–135, [18]), running on lme4 (v.1.1–7, [19]), using the R statistical package (R Development Core Team, R version 3.1.2 (Pumpkin Helmet)). The model contained the three within-participant factors as fixed effects to predict participant’s decisions to play or pass on a 50–50 mixed gamble (binary variable). To account for the repeated-measures and nested nature of the data, we included random adjustments to the fixed intercept (“random intercept”) for participant in the model. To allow individual variation with respect to the fixed effects, we included within-unit random slopes for context type, feedback context, gamble type, and an interaction term between feedback and gamble type within participant, allowing random correlations among random effects (e.g., [20, 21, 22]). P values were determined using Likelihood Ratio Tests as implemented in the *mixed*() function [18], and for all post-hoc pairwise multiple comparisons we used the general linear hypothesis test (*glht*) function of the multcomp package (v.1.3–8), suitable for generalized linear mixed effects models [23]. Reported means are the least-squares means and confidence intervals (CI) are set at 95%, obtained using the *lsmeans* function of the lsmeans package (v.2.13, [24]). For the data, see S1 Data File.

## Results

Our main question of interest was to examine individuals’ inconsistent risk preferences for positive and negative outcomes (feedback context) by different types of feedback (context type); monetary gains and losses (M), performance success and failure (P), and performance success and failure in combination with monetary gain and loss (MP). Supporting previous findings [2, 10, 11, 25] we found that participants’ risk preferences were sensitive to positive and negative feedback contexts (*χ* ^*2*^ (1) = 16.91, *p* < .001). Participants took more risks after previously experiencing negative feedback as compared to previously experiencing positive feedback, irrespective of the context type (*M*_negative_ = 0.396, *CI* = [0.264, 0.546], *M*_positive_ = 0.224, *CI* = [0.122, 0.376]).

**Fig 2:**
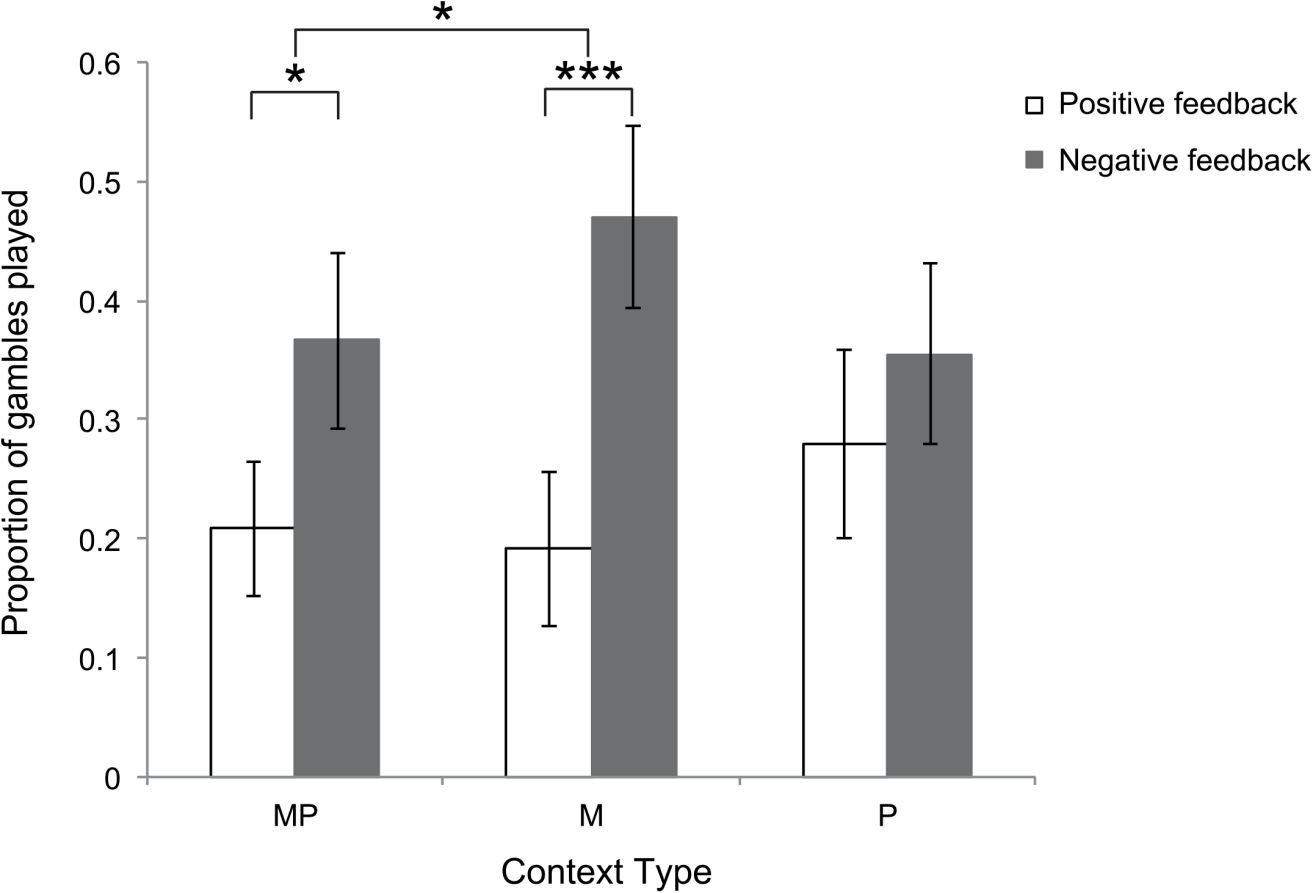
Risk-taking following different types of positive and negative feedback contexts. MP = performance feedback including monetary gain/loss, M = monetary gain/loss, P = performance success/failure. Error bars represent standard errors of the mean. * *p* < .05, *** *p* < .001.

In terms of our primary question of interest, context type (M, P, MP) significantly influenced participants’ risky behaviour following positive/negative feedback (*χ* ^*2*^ (2) = 28.62, *p* < .001). Experiencing incidental monetary gains and losses shifts subsequent risk preferences most strongly; *M*_M loss_ = 0.471, *CI* = [0.332, 0.614], *M*_M gain_ = 0.192, *CI* = [0.103, 0.327], z = 8.998, *p* < .001). Experiencing performance-based monetary gains and losses also caused a significant shift in risk preferences, *M*_MP loss_ = 0.366, *CI* = [0.232, 0.525], and *M*_MP gain_ = 0.209, *CI* = [0.109, 0.363], though significantly weaker than for monetary gain/loss feedback (*χ* ^*2*^ (2) = 6.31 *p* = .01). However, risk-taking behaviour following performance-based success did not significantly differ to that following failure (*M*_P failure_ = 0.355, *CI* = [0.222, 0.514], *M*_P success_ = 0.280, *CI* = [0.152, 0.457], z = 2.160, *p* = 0.257) (Fig 2).

Examining the data more closely, we find that participants take more risk after a ‘pure’ monetary loss than after experiencing a monetary loss based on performance failure (Loss: M–MP, *z* = 3.436, *p* = .008), and as compared to performance failure only (Loss: M–P, *z* = 3.804, *p* = .002). In the positive feedback context we find a different pattern, that is, participants took more risk after receiving success performance-based feedback than after a ‘pure’ monetary gain (Gain: P–M, *z* = 3.246, *p* = .015), but not more than success performance-based feedback in combination with monetary gain (Gain: P–MP, *z* = 2.545, *p* = 0.111). There was no significant difference in risk-taking whether participants received a ‘pure’ monetary gain or whether participants received a monetary gain in combination with success feedback (Gain: M—MP, *z* = -0.735, *p* = 0.978), and also not after receiving failure performance feedback, with or without a monetary loss (Loss: MP–P, *z* = 0.320, *p* = 1.0). Overall participants did not take different proportion of risk following the different context types (*M*_MP_ = 0.281, *CI* = [0.165, 0.435] *M*_M_ = 0.315, *CI* = [0.198, 0.461] *M*_P_ = 0.316, *CI* = [0.189, 0.478], *χ* ^*2*^ (2) = 2.48, *p* = 0.289).

Participants were sensitive to the expected values of the gambles, with the greatest willingness to play the positive EV gamble and the lowest willingness to play the negative EV gamble (*M*_+EV_ = 0.768, *CI* = [0.581, 0.888], *M*_0EV_ = 0.295, *CI* = [0.161, 0.478], *M*-_EV_ = 0.056, *CI* = [0.029, 0.107], *χ* ^*2*^ (2) = 44.50, *p* < .001). The expected value of the gamble also affected risk preferences differently following positive and negative feedback (Feedback context x EV gamble: *χ* ^*2*^ (2) = 14.40, *p* = .001). For all expected values, participants gambled more following negative compared to positive feedback. The type of context (i.e. M, P, and MP) did not affect risk preferences for gambles with different expected values (*χ* ^*2*^ (2) = 2.45, *p* = .654).

## Discussion

In this study, we investigated whether context-dependent shifts in financial risk preferences are driven primarily by the delivery of monetary gains and losses, or are rather due to the receipt of performance-based success and failure feedback. To examine this, we directly induced both performance and monetary contexts prior to, and independent of, choices to either play or pass identical mixed gambles. Firstly, we replicated the framing effect on behaviour for performance-based monetary gains and losses that we observed in our previous work [10], namely that prior losses induced greater gambling than prior gains. Secondly, incidental monetary gains/losses, that is, those not related to the task performance itself, impacted risk preferences in a similar fashion to performance-based monetary gains and losses. Thirdly, performance-based feedback of success and failure, without associated monetary rewards or punishments, did not differentially affect individuals risk preferences for the subsequent gambles. Fourthly, we found that incidental, randomly distributed, monetary gains and losses caused the largest shift in risk preferences, significantly more so than performance-based monetary gains and losses. Finally, across all context types people were sensitive to the expected values of the gambles, accepting the highest expected value gambles more than the zero and negative expected value gambles. Moreover, the typical framing effect was observed for all gamble types.

The finding that risk preferences are differentially susceptible to prior positive and negative contexts, though only when these preceding contexts involve monetary gains and losses, is consistent with previous research [7, 8], and can potentially be attributed to loss aversion [11]. Loss aversion, the empirically observed phenomenon whereby losses loom larger than equivalent gains, can lead players to more likely pass on a mixed gamble which includes the possibility of losing money. However, in a situation in which we have already just lost money, either through random chance or due to poor performance, a mixed gamble that offers a chance to redeem this loss becomes substantially more tempting. Taken together, our behavioural tendency to want to avoid a loss is one possible explanatory process for the observed findings here, that of a typical shift in risk preferences for mixed gambles following unrelated monetary gains/losses of any type.

One possible explanation for why gains and losses could impact subsequent gambling preference is that this feedback might lead to differences in experienced mood [16] that might in turn influence preferences for risk. Prior studies examining the effect of positive and negative mood on risk preferences have indeed found that incidental positive mood influenced subsequent preferences for high risk gambles [13], and enhanced framing effects [14]. In the current study however, we used a condition whereby performance feedback was provided in the absence of monetary gains and losses, and notably we did not find a difference in risk preferences following successful or failed performance. Given that we might expect the feedback alone to impact mood, the lack of a contextual effect here suggests that potential mood effects seem unlikely to underlie the observed shifts in risk-taking. We cannot rule out that receiving a monetary outcome through random chance (i.e. M condition) may have resulted in a stronger incidental mood experience as compared to the performance-based conditions. If a monetary outcome would indeed result in a stronger incidental mood, we would have expected to see, in line with prior work [13, 14], an increase in risk-taking following a monetary gain as compared to a good performance. It should be noted that the during the P and MP conditions participants did not know in advance whether they would receive only performance feedback or whether they would receive performance feedback with an associated monetary reward or punishment. This random presentation may have resulted in a weaker effect of feedback on subsequent risk preferences when receiving only performance-based (P) feedback and a stronger effect in the MP trials, depending on the participant’s expectation. However, the differences in risk-taking in the MP trials are similar to the difference we observed in our previous study [10], one that only employed MP trials, indicating that the randomization may also not have led to an effect on mood.

The success and failure feedback provided here was based on participants’ actual performance, which is different to experiencing incidental monetary gains and losses. Performance feedback therefore, could be too unrelated to the subsequent gamble decision to be integrated into the valuation of the subsequent choice. Monetary gains and losses are compatible with the (monetary) outcome of mixed gambles, with performance success and failure providing a less compelling link, leading to a weaker impact on behaviour for the latter. This is consistent with the compatibility effect [26], which suggests that outcomes that are compatible with the output of choice are given more weight. Nevertheless, when comparing the different context types separately for positive and negative feedback, we find that feedback of success does indeed increase subsequent risk-taking, but only compared to pure monetary gains and not compared to performance-based monetary gains. One potential explanation for this increased risk-taking following successful performance is that the player actually has nothing to lose, as compared to receiving a monetary gain through random chance. The latter may motivate the player to want to consolidate this random gain, therefore showing a stronger risk aversion for the mixed gamble. For both pure monetary gains as well as performance-based monetary gains, we found a similar degree of risk aversion for mixed gambles.

The finding that behavioural framing effects for incidental monetary gains and losses were stronger than performance-based monetary gains and losses, suggests that these framing effects are primarily driven by the receipt of monetary outcomes, and not, or at least less so, by success and failure. Additional feedback about performance success or failure may therefore reduce loss aversion. It is likely that receiving a ‘random’ monetary loss (i.e. M condition), that is, without it being their “fault”, may in fact feel like a subjectively stronger punishment. Receiving such an incidental monetary loss may therefore enhance the willingness to try to retrieve this money via a subsequent gamble, a speculation that the current data supports. Conversely, incurring a loss based on a bad actual performance might feel more justified, and therefore might result in weaker framing effects.

The differential behavioural effects we found for pure performance-based success/failure, and performance-based success/failure in combination with monetary gains/losses, have also been observed with functional neuroimaging data. Monetary reward and punishments exhibited more enhanced activity in overlapping brain regions compared to pure performance-based success and failure [27, 28, 29, 30]. These areas have also been associated with anticipation of reward and financial risk-taking [31, 32, 33, 34]. The stronger response in the reward and punishment-related network in the brain to monetary feedback relative to performance-based feedback supports our finding that monetary feedback influences subsequent choice more strongly then monetary feedback confounded with performance-based feedback.

One other potential explanation for the differences we observe across contexts is that a difference in amount of effort might be required by the varying context type conditions, leading to an ego-depletion effect and subsequently to alterations in risk-taking. In comparison to the M condition, where participants simply received monetary gains and losses, the other two performance conditions required some effort, namely the estimation of a one second temporal duration. However, we believe this effort to be minimal, and would not expect that this small effort would be sufficient to lead to ego depletion. Further, previous studies have reported a positive relationship between cognitive control and the capacity to resist framing biases [35, 36]. If participants’ resources indeed were depleted during the P and MP trials due to the efforts expended on the time-estimation task, we might therefore actually expect the opposite behavioural pattern than was found. Nevertheless, future studies could usefully explore whether ego depletion could have an effect on the experiences of feedback and subsequent risk preferences.

The current findings indicate that participants’ risk preferences in case of monetary gambles are very susceptible to the provision of prior monetary amounts, but that this effect does not easily extend to non-monetary (or non-compatible) contexts. This observed context-specificity seems to reflect a compatibility effect [26], namely that preceding monetary gain/loss outcomes (rather than success or failure by itself) are easier to integrate into the subsequent choice process to play/pass a gamble consisting of monetary outcomes. Hence, positively or negatively valenced contexts do not affect risk preferences in a consistent way. Future studies could provide more insight into how these different context types may affect our valuation of risky choice by examining the underlying psychological and neural differences mediated by different reward and punishment contexts. Furthermore, it would be interesting to examining whether effects of different context types exist for risk choices containing non-monetary outcomes, e.g. health-related decisions, to gain a better view of cross-context effects on risk.

## Conclusion

In the current study we showed that risky shifts following a prior positive and negative feedback are differentially affected by contexts that are based on money or on individuals’ performance. The risky shift (i.e. framing effect) is primarily driven by monetary gains and losses, and less so due to prior success or failure. In other words, simply seeing the results of a lottery in which participants won or lost immediately preceding a monetary mixed gamble, without performing any task, enhanced the current framing effect. On the contrary, when participants were informed whether they had been successful or unsuccessful in the previous task, risk preferences for mixed gambles became more consistent, and thus, framing disappeared. These results highlight the strong, specific, context-dependency of risk preferences and choices, and suggests that positive or negative valence or mood *per se* do not influence this, but rather our aversion to losses appears to be the fundamental process explaining the observed inconsistency in risk preferences following gain and loss outcomes.

## Supporting Information

S1 Data File Response: dependent variable, 1 = decision to play gamble, 0 = pass on gamble; condition: context type, M = Money only, P = Performance only, MP = Money and Performance feedback; feedback: feedback following the cue; gambletype: expected value of the gamble, 4 = + EV, 5 = 0 EV, 6 = —EV; fdPriorGamble: outcome of prior gamble; Fdcond: feedback x condition variable for post hoc tests; FdGT: feedback x gambletype variable for post hoc tests (XLS)
